# Supplementary figures and images for: Identification of potent inhibitors of HDAC2 from herbal products for the treatment of colon cancer: Molecular docking, molecular dynamics simulation, MM/GBSA calculations, DFT studies, and pharmacokinetic analysis
Source: PLoS One. 2024 Jul 22;19(7):e0307501. doi: 10.1371/journal.pone.0307501 (PMC11262678; doi:10.1371/journal.pone.0307501)

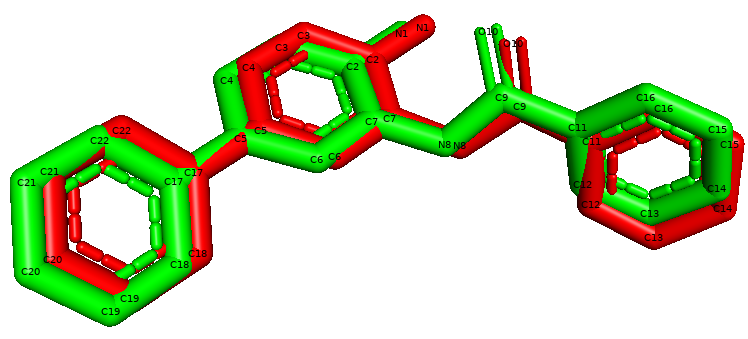

Supplement: S1 Fig — (TIFF) [file pone.0307501.s001.tiff]

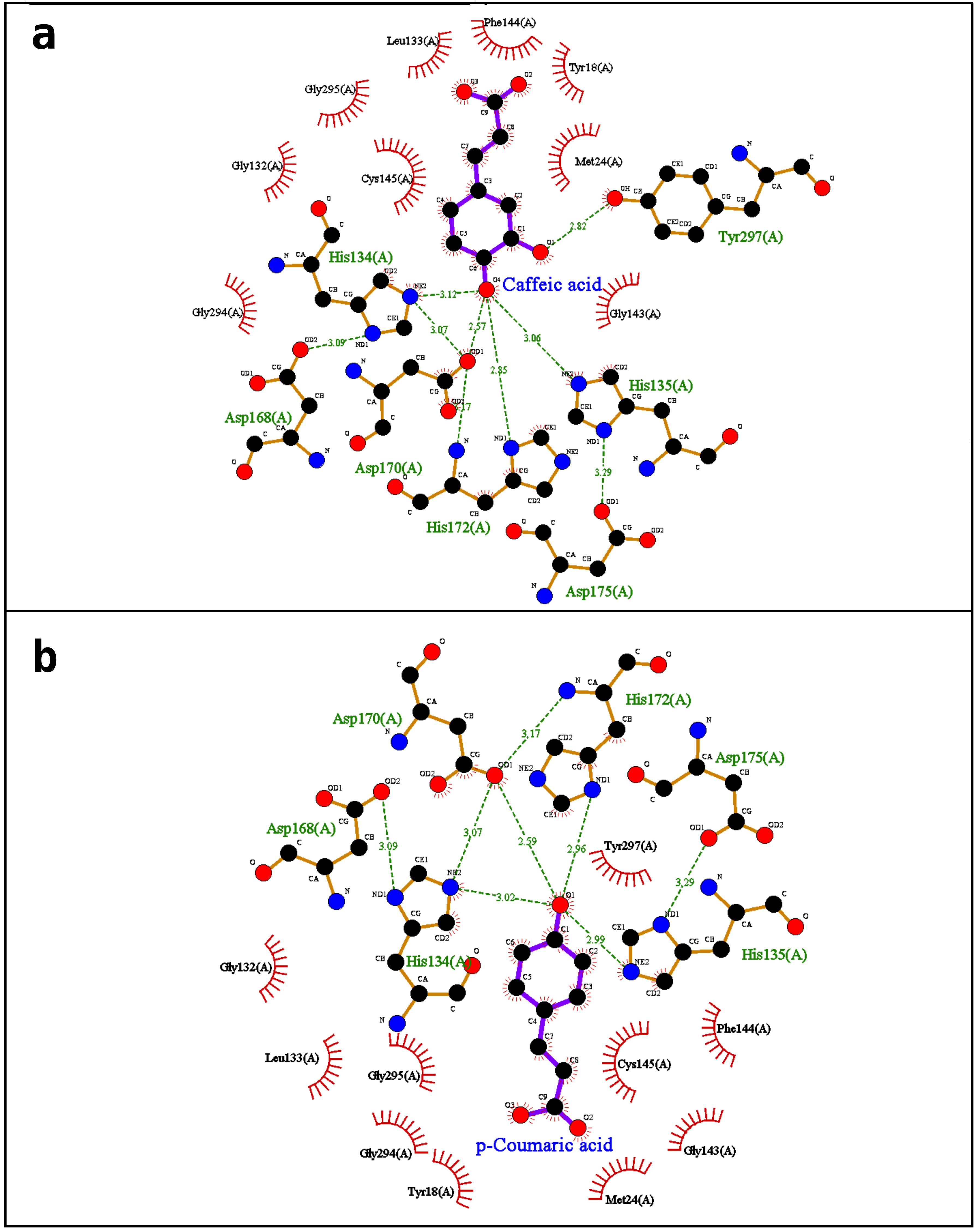

Supplement: S2 Fig — (a) CA-HDAC2 and (b) pCA-HDAC2 complexes prepared by LigPlot+ V.2.2 program. (TIFF) [file pone.0307501.s002.tiff]

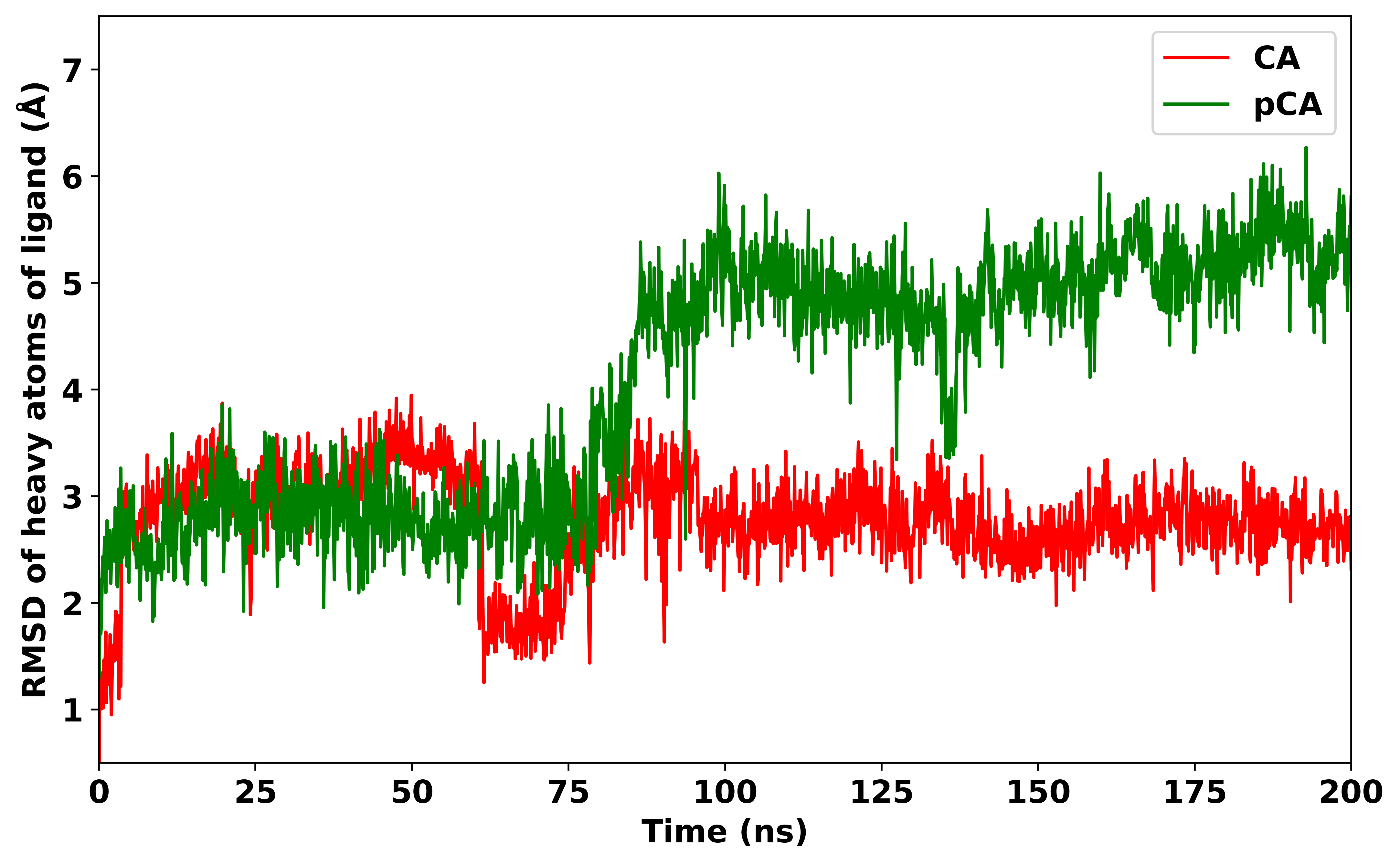

Supplement: S3 Fig — (TIFF) [file pone.0307501.s003.tiff]

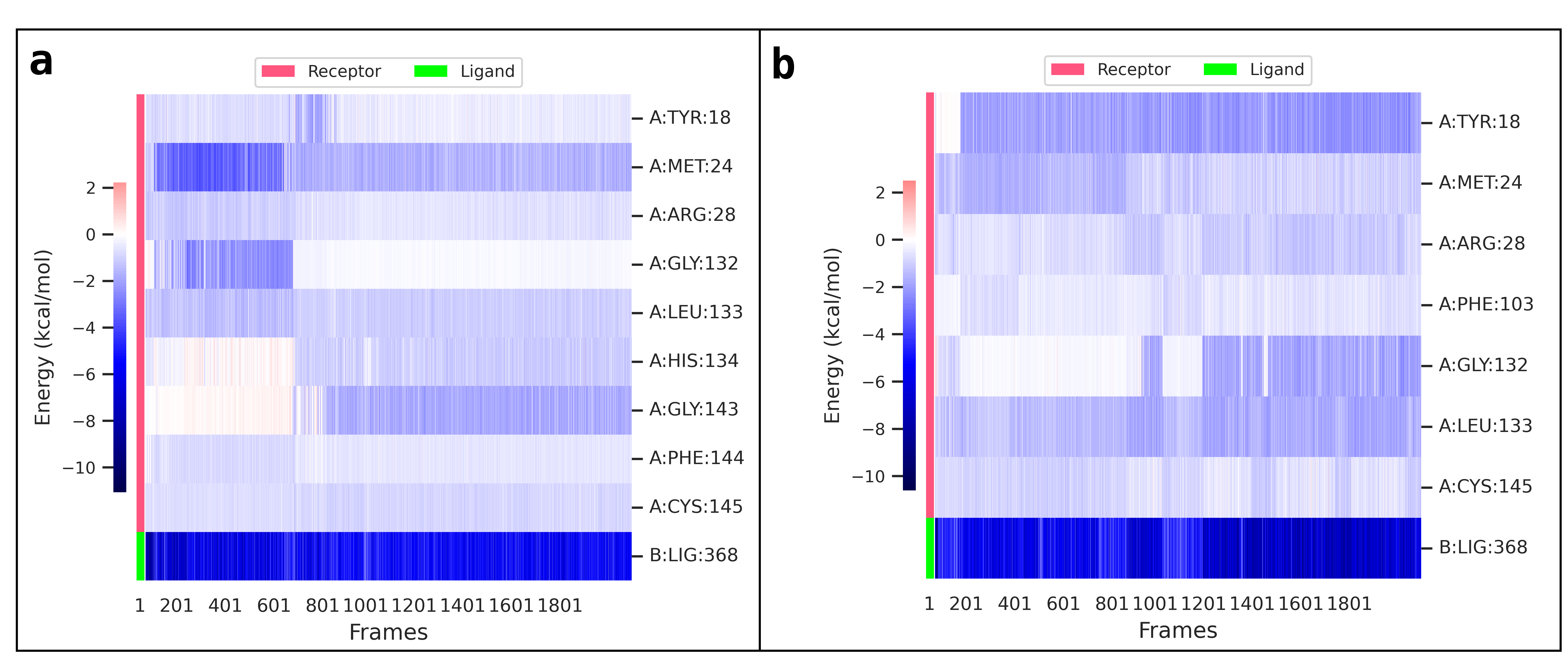

Supplement: S4 Fig — (a) CA-HDAC2 and (b) pCA-HDAC2 complexes. (TIFF) [file pone.0307501.s004.tiff]

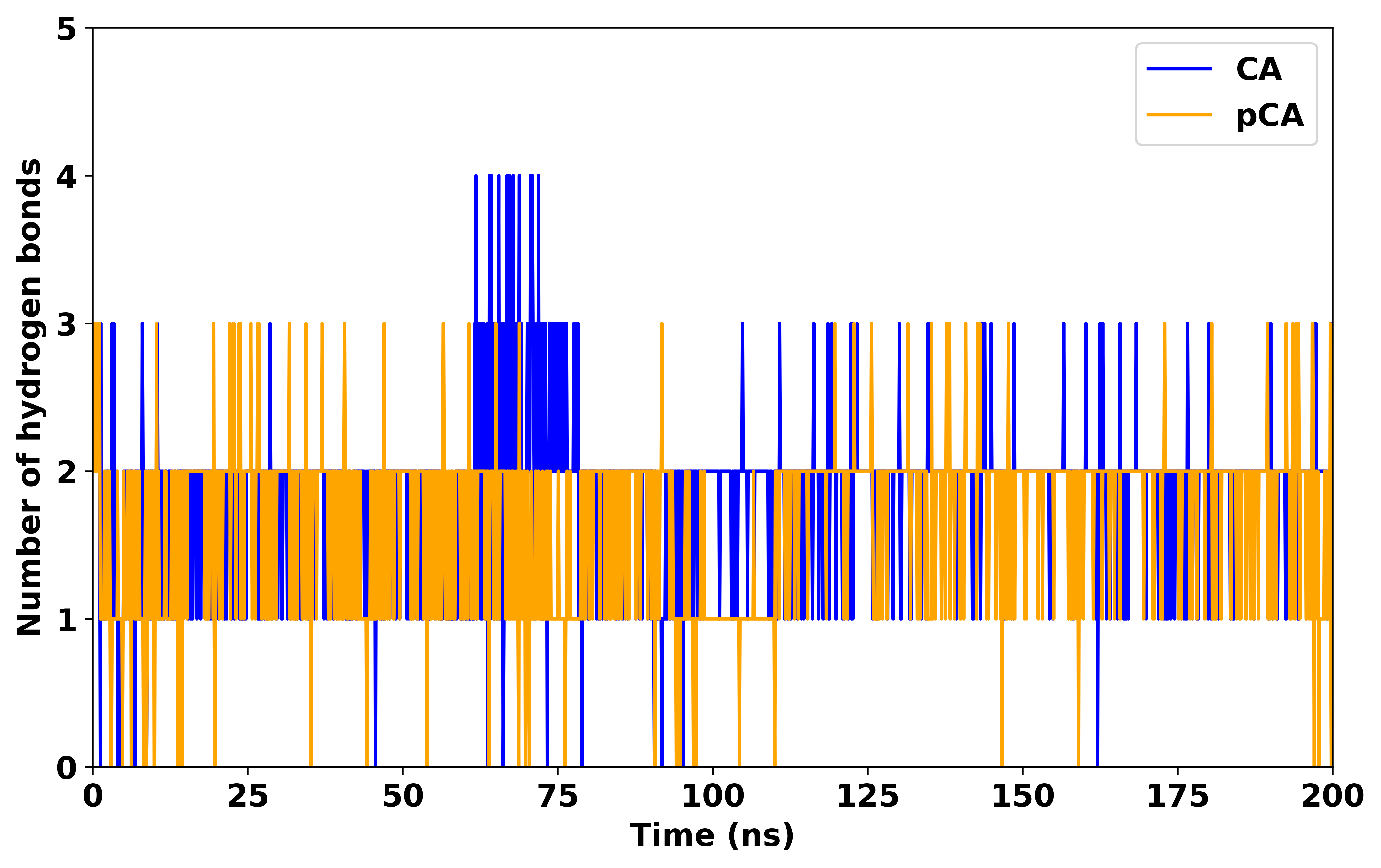

Supplement: S5 Fig — (TIFF) [file pone.0307501.s005.tiff]

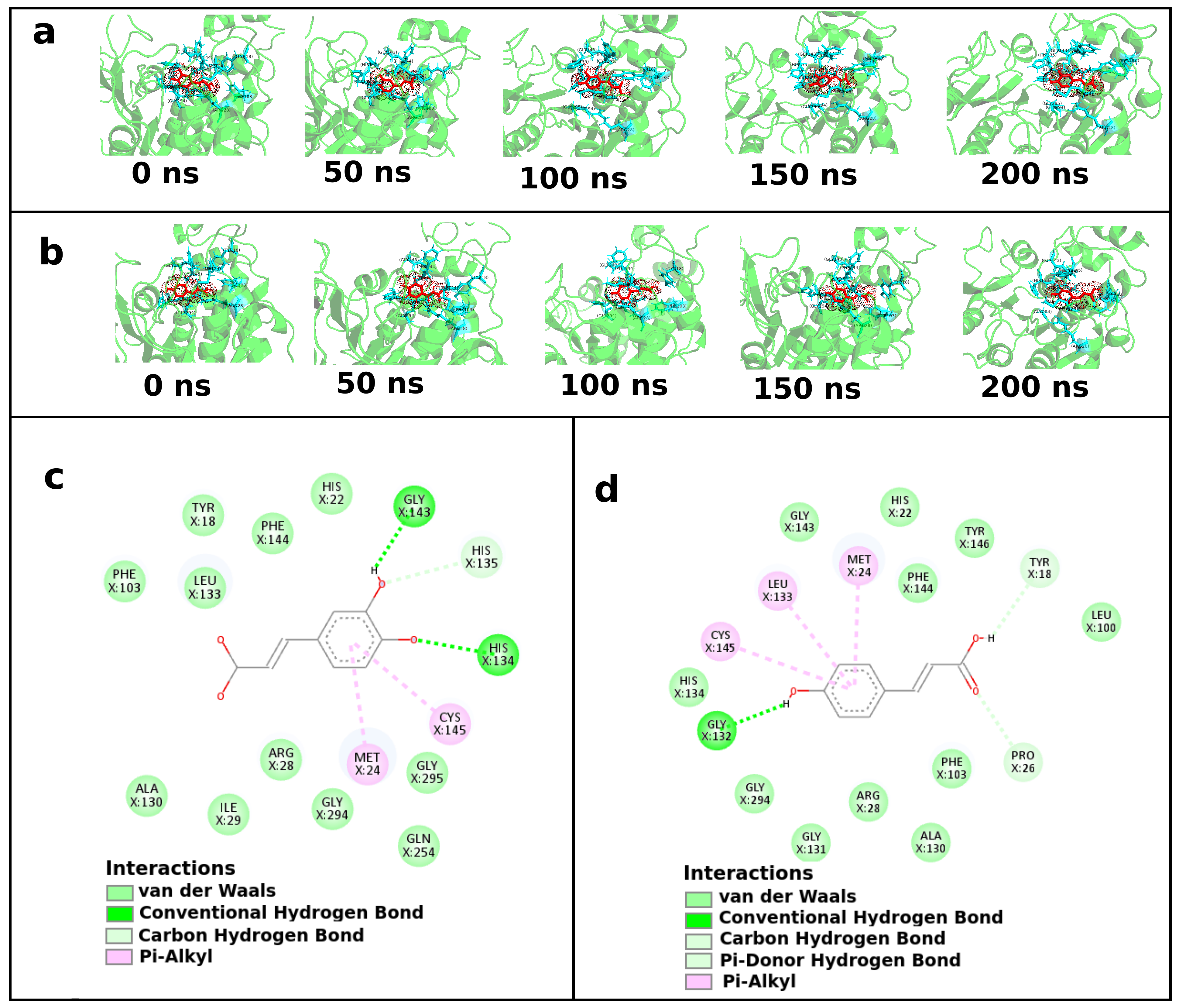

Supplement: S6 Fig — (a) CA-HDAC2 and (b) pCA-HDAC2 complexes. The 2D diagrams illustrate interactions of the compounds with active amino acid residues at the 2000th frame of MD simulations for (c) CA-HDAC2 and (d) pCA-HDAC2 complexes. (TIFF) [file pone.0307501.s006.tiff]

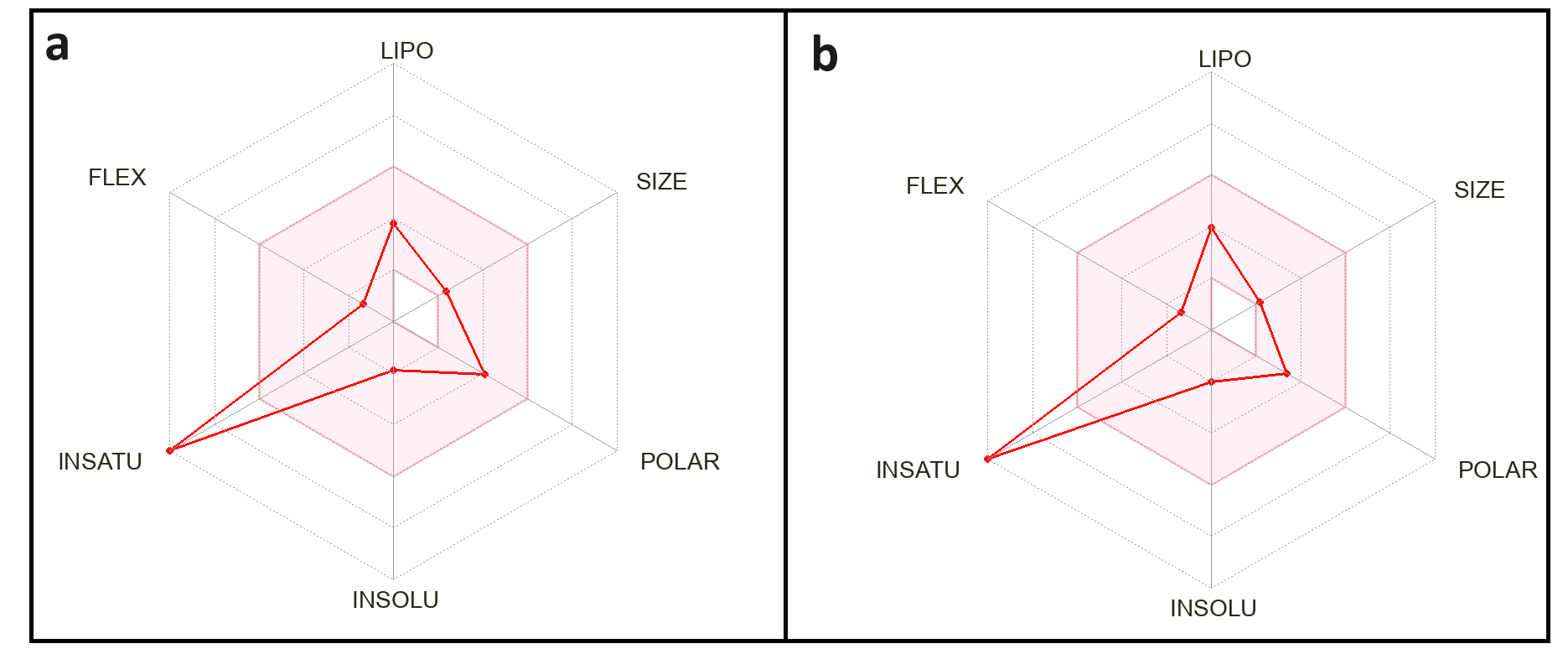

Supplement: S7 Fig — (a) CA molecule and (b) pCA molecule. (TIFF) [file pone.0307501.s007.tiff]

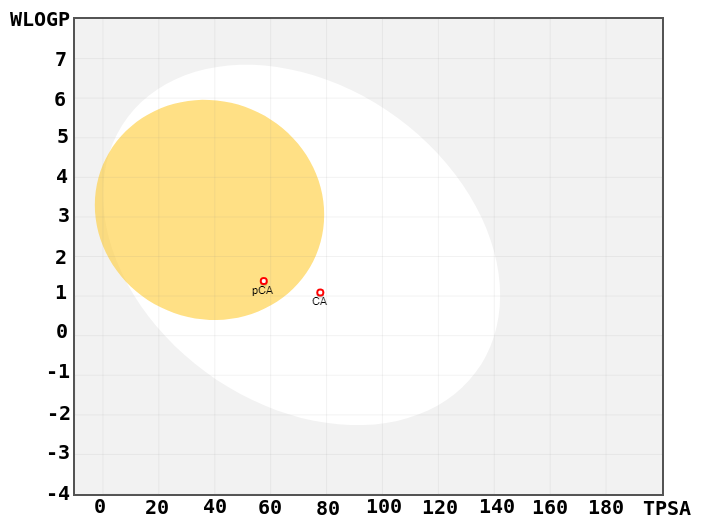

Supplement: S8 Fig — (TIFF) [file pone.0307501.s008.tiff]
