# Supplementary material for: Identification of potent inhibitors of HDAC2 from herbal products for the treatment of colon cancer: Molecular docking, molecular dynamics simulation, MM/GBSA calculations, DFT studies, and pharmacokinetic analysis
Source: PLoS One. 2024 Jul 22;19(7):e0307501. doi: 10.1371/journal.pone.0307501 (PMC11262678; doi:10.1371/journal.pone.0307501)
Supplement: S1 Table — (PDF) [file pone.0307501.s009.pdf]

S1 Table: Binding energy of some selected biomolecules for virtual screening using pyRx

Biomolecules	Binding Energy (kcal/mol)
Caffeic acid	-7.0
p-Coumaric acid	-6.7
Epipodophyllotoxin	-6.7
Ferulic acid	-6.7
Parthenolide	-6.6
Seliciclib	-6.5
Helenalin	-6.5
Arctigenin	-6.4
Cinnamyl alcohol	-6.3
Cinnamaldehyde	-6.1
Sinapinic acid	-6.1
Fosbretabulin	-5.8
Noscapine	-5.7
Nulforaphane	-4.5
Allicin	-4.0
Diallyl sulfide	-3.7
Allyl mercaptan	-2.6
