# Supplementary material for: Identification of potent inhibitors of HDAC2 from herbal products for the treatment of colon cancer: Molecular docking, molecular dynamics simulation, MM/GBSA calculations, DFT studies, and pharmacokinetic analysis
Source: PLoS One. 2024 Jul 22;19(7):e0307501. doi: 10.1371/journal.pone.0307501 (PMC11262678; doi:10.1371/journal.pone.0307501)
Supplement: S2 Table — (PDF) [file pone.0307501.s010.pdf]

S2 Table: Second order perturbation theory analysis of Fock matrix on NBO basis for CA molecule using B3LYP/6-311++G(d,p)

Donor NBO (i)	Type	Acceptor NBO (j)	Type	E(2) kcal/mol	E(j)-E(i) a.u.	F(i,j) a.u.
O3	LP(2)	O4-C13	σ^*	41.24	0.35	0.11
O4	LP(2)	O3-C13	σ	33.61	0.60	0.13
O1	LP(2)	C6-C8	σ^*	27.31	0.35	0.09
O2	LP(2)	C9-C10	σ^*	24.67	0.36	0.09
C11-C12	π	O4-C13	π^*	21.68	0.29	0.07
C5-C7	π	C9-C10	π^*	21.10	0.26	0.07
C6-C8	π	C9-C10	π^*	20.96	0.28	0.07
C5-C7	π	C6-C8	π^*	19.73	0.27	0.07
C9-C10	π	C5-C7	π^*	19.44	0.30	0.07
C6-C8	π	C5-C7	π^*	19.14	0.29	0.07
C9-C10	π	C6-C8	π^*	17.94	0.30	0.07
C6-H14	π	C11-C12	π^*	16.90	0.70	0.10
C5-C7	π	C11-C12	π^*	14.16	0.29	0.06
C11-C12	π	C5-C7	π^*	10.26	0.30	0.05
O1-C8	π	C10-H16	π^*	6.47	1.24	0.08
C5-C6	π	C7-H15	π^*	6.07	1.18	0.08
C11-C12	π	C11-H17	π^*	5.68	0.95	0.07
O1-C8	π	C5-C7	π^*	5.47	1.15	0.07
C12-H18	π	C11-C12	π^*	4.91	1.07	0.06
C7-C10	π	O2-C9	π^*	4.81	1.03	0.06
C6-H14	π	C5-C7	π^*	4.60	1.31	0.07
C12-C13	π	C11-C12	π^*	4.34	1.08	0.06
C9-C10	π	C7-C10	π^*	4.33	0.97	0.06
C7-C10	π	C9-C10	π^*	4.23	1.09	0.06
C11-C12	π	O3-C13	π^*	4.19	1.17	0.06
C6-C8	π	O1-H19	π^*	4.15	1.14	0.06
O3-C13	π	C11-C12	π^*	4.13	1.26	0.06
C5-C7	π	C11-H17	π^*	3.94	1.29	0.06
O1-C8	π	C9-C10	π^*	3.93	1.27	0.06
C7-C10	π	C10-H16	π^*	3.85	1.06	0.06
O1-C8	π	C5-C6	π^*	3.82	1.04	0.06
O2-C9	π	C6-C8	π^*	3.82	1.25	0.06
C11-C12	π	C12-H18	π^*	3.78	1.07	0.06
C12-C13	π	O4-C13	π^*	3.60	1.21	0.06
C11-C12	π	C12-C13	π^*	3.53	1.19	0.06
C12-H18	π	C11-C12	π^*	3.51	1.10	0.06
O2-C9	π	C6-C8	π^*	3.46	1.26	0.06
C11-C12	π	O3-C13	π^*	3.45	1.26	0.06
C6-C8	π	O1-H19	π^*	3.42	1.07	0.05
O3-C13	π	C11-C12	π^*	3.27	1.31	0.06
C5-C7	π	C11-H17	π^*	3.24	1.08	0.05
O1-C8	π	C10-H16	π^*	3.22	0.42	0.03
C7-C10	π	C9-C10	π^*	3.13	1.27	0.06
O2-C9	π	C7-C10	π^*	3.11	1.04	0.05

LP = lone pair, (j) acceptor, (i) donor, $E(2)$ represents the stabilization energy. $F(i,j)$ is the Fock matrix element between donor and acceptor orbitals i and j, and $E(j) - E(i)$ is the energy difference between donor and acceptor orbitals i and j NBO orbitals.

Donor NBO (i)	Type	Acceptor NBO (j)	Type	E(2) kcal/mol	E(j)-E(i) a.u.	F(i,j) a.u.
O2-C9	π	C9-C10	π^*	3.09	1.21	0.06
C5-C7	π	C7-C10	π^*	3.05	1.32	0.06
C5-C7	π	C6-C8	π^*	3.02	1.27	0.06
C8-C9	π	O1-C8	π^*	3.02	1.26	0.06
C11-C12	π	C6-C8	π^*	3.01	1.31	0.06
C5-C11	π	C5-C6	π^*	2.92	1.24	0.05
C5-C6	π	C5-C11	π^*	2.91	1.19	0.05
C5-C11	π	C5-C7	π^*	2.91	1.23	0.05
C6-C8	π	C5-C6	π^*	2.89	1.28	0.05
C11-C12	π	C11-H17	π^*	2.74	1.14	0.05
C6-C8	π	O2-C9	π^*	2.61	1.26	0.05
C11-C12	π	C5-C11	π^*	2.52	1.11	0.05
C5-C7	π	C7-C10	π^*	2.46	1.13	0.05
C5-C11	π	C11-C12	π^*	2.37	1.13	0.05
C5-C7	π	C7-C10	π^*	2.37	1.23	0.05
C5-C11	π	C12-C13	π^*	2.23	1.19	0.05
C11-C12	π	O4-C13	π^*	2.17	1.32	0.05
C5-C6	π	C11-C12	π^*	2.14	1.17	0.04
C11-C12	π	C12-C13	π^*	2.12	1.52	0.05
C11-C12	π	C5-C7	π^*	2.11	1.14	0.04
C5-C11	π	C12-C13	π^*	2.06	1.16	0.04
C5-C7	π	O1-C8	π^*	2.05	1.07	0.04
C11-C12	π	C6-C8	π^*	2.00	1.34	0.05
C5-C6	π	C5-C11	π^*	1.98	1.11	0.04
C5-C11	π	C7-C10	π^*	1.97	1.31	0.04
C5-C7	π	C10-H16	π^*	1.89	1.25	0.04
C5-C6	π	C7-H15	π^*	1.87	1.23	0.04
C5-C7	π	C6-H14	π^*	1.87	1.11	0.04
C5-C11	π	C5-C6	π^*	1.76	1.18	0.04
C5-C7	π	C5-C11	π^*	1.74	1.29	0.04
C5-C11	π	C5-C7	π^*	1.73	1.19	0.04
C6-C8	π	C5-C6	π^*	1.68	1.18	0.04
C11-C12	π	C11-C12	π^*	1.54	1.50	0.04
C5-C6	π	C5-C6	π^*	1.54	1.09	0.04
C11-C12	π	C12-C13	π^*	1.45	1.12	0.04
C12-C13	π	C11-C12	π^*	1.43	1.56	0.04
C11-C12	π	C5-C6	π^*	1.37	1.14	0.04
C5-C11	π	C5-C11	π^*	1.36	1.47	0.04
C5-C7	π	C5-C7	π^*	1.34	1.13	0.04
C5-C6	π	C5-C6	π^*	1.30	1.48	0.04
C5-C7	π	C6-C8	π^*	1.30	1.03	0.03
C5-C11	π	C6-C8	π^*	1.18	1.13	0.03
C6-C8	π	O2-C9	π^*	1.16	1.19	0.03
C11-C12	π	C11-H17	π^*	1.11	1.49	0.04
C5-C11	π	C7-C10	π^*	1.09	1.16	0.03
C5-C7	π	C11-H17	π^*	1.07	1.48	0.04
C6-C8	π	C11-C12	π^*	1.05	1.51	0.04
C8-C9	π	O1-C8	π^*	1.01	1.13	0.03
