# Supplementary material for: Identification of potent inhibitors of HDAC2 from herbal products for the treatment of colon cancer: Molecular docking, molecular dynamics simulation, MM/GBSA calculations, DFT studies, and pharmacokinetic analysis
Source: PLoS One. 2024 Jul 22;19(7):e0307501. doi: 10.1371/journal.pone.0307501 (PMC11262678; doi:10.1371/journal.pone.0307501)
Supplement: S3 Table — (PDF) [file pone.0307501.s011.pdf]

S3 Table: Second order perturbation theory analysis of Fock matrix on NBO basis for pCA molecule using B3LYP/6-311++G(d,p)

Donor NBO (i)	Type	Acceptor NBO (j)	Type	E(2) kcal/mol	E(j)-E(i) a.u.	F(i,j) a.u.
O2	LP(2)	O3-C12	$\pi*$	41.19	0.35	0.11
O3	LP(2)	O2-C12	$\sigma*$	33.56	0.60	0.13
O1	LP(2)	C8-C9	$\pi*$	27.55	0.35	0.09
C8-C9	π	C4-C6	$\pi*$	24.48	0.29	0.08
C4-C6	π	C5-C7	$\pi*$	22.57	0.27	0.07
C5-C7	π	C8-C9	$\pi*$	22.26	0.28	0.07
C10-C11	π	O3-C12	$\pi*$	21.84	0.29	0.07
C4-C6	π	C8-C9	$\pi*$	18.76	0.27	0.06
C4-C6	π	C10-C11	$\pi*$	18.55	0.29	0.07
O3	LP(2)	C11-C12	$\sigma*$	16.85	0.70	0.10
C8-C9	π	C5-C7	$\pi*$	16.59	0.28	0.06
C5-C7	π	C4-C6	$\pi*$	15.48	0.29	0.06
C10-C11	π	C4-C6	$\pi*$	10.85	0.30	0.05
O2	LP(1)	O3-C12	$\sigma*$	6.46	1.24	0.08
O1	LP(1)	C7-C9	$\sigma*$	6.06	1.16	0.08
C10-H17	σ	C11-H18	$\sigma*$	5.67	0.95	0.07
C10-H17	σ	C4-C5	$\sigma*$	5.00	1.07	0.07
C6-H14	σ	C4-C5	$\sigma*$	4.36	1.08	0.06
C11-H18	σ	C10-H17	$\sigma*$	4.33	0.97	0.06
O1-H19	σ	C8-C9	$\sigma*$	4.32	1.31	0.07
C5-C7	σ	O1-C9	$\sigma*$	4.20	1.06	0.06
C11-C12	σ	C4-C10	$\sigma*$	4.20	1.17	0.06
C5-H13	σ	C4-C6	$\sigma*$	4.18	1.08	0.06
C11-H18	σ	O3-C12	$\sigma*$	4.16	1.14	0.06
C8-H16	σ	C7-C9	$\sigma*$	4.09	1.07	0.06
C7-C9	σ	C8-C9	$\sigma*$	4.02	1.27	0.06
C7-H15	σ	C8-C9	$\sigma*$	3.87	1.09	0.06
C8-C9	σ	C7-C9	$\sigma*$	3.79	1.26	0.06
C8-H16	σ	C4-C6	$\sigma*$	3.76	1.08	0.06
C6-C8	σ	O1-C9	$\sigma*$	3.75	1.05	0.06
C7-H15	σ	C4-C5	$\sigma*$	3.68	1.10	0.06
C5-H13	σ	C7-C9	$\sigma*$	3.58	1.07	0.06
O2-H20	σ	C11-C12	$\sigma*$	3.53	1.19	0.06
C5-C7	σ	C4-C10	$\sigma*$	3.52	1.21	0.06
C4-C5	σ	C4-C6	$\sigma*$	3.51	1.25	0.06
C4-C6	σ	C4-C5	$\sigma*$	3.50	1.25	0.06
C6-H14	σ	C8-C9	$\sigma*$	3.40	1.08	0.05
C6-C8	σ	C4-C10	$\sigma*$	3.28	1.21	0.06
O3-C12	π	C10-C11	$\pi*$	3.20	0.42	0.03
C5-C7	σ	C4-C5	$\sigma*$	3.18	1.27	0.06
C4-C10	σ	C10-C11	$\sigma*$	3.11	1.32	0.06
C4-C5	σ	C5-C7	$\sigma*$	3.02	1.28	0.06
C7-C9	σ	C5-C7	$\sigma*$	2.99	1.30	0.06
C6-C8	σ	C4-C6	$\sigma*$	2.98	1.27	0.06
C4-C5	σ	C4-C10	$\sigma*$	2.95	1.19	0.05

Donor NBO (i)	Type	Acceptor NBO (j)	Type	E(2) kcal/mol	E(j)-E(i) a.u.	F(i,j) a.u.
C10-C11	σ	C4-C10	$\sigma*$	2.94	1.24	0.05
C4-C10	σ	C4-C5	$\sigma*$	2.93	1.23	0.05
C5-C7	σ	C7-C9	$\sigma*$	2.90	1.26	0.05
O3	LP(1)	C11-C12	$\sigma*$	2.74	1.14	0.05
C4-C6	σ	C6-C8	$\sigma*$	2.72	1.28	0.05
C8-C9	σ	C6-C8	$\sigma*$	2.69	1.30	0.05
C6-C8	σ	C8-C9	$\sigma*$	2.66	1.26	0.05
C4-C6	σ	C5-H13	$\sigma*$	2.51	1.13	0.05
C4-C10	σ	C4-C6	$\sigma*$	2.50	1.23	0.05
C4-C6	σ	C4-C10	$\sigma*$	2.47	1.19	0.05
C4-C5	σ	C7-H15	$\sigma*$	2.42	1.11	0.05
C8-C9	σ	C6-H14	$\sigma*$	2.33	1.15	0.05
C4-C5	σ	C6-H14	$\sigma*$	2.29	1.13	0.05
C4-C6	σ	C8-H16	$\sigma*$	2.29	1.13	0.05
C8-C9	σ	C7-H15	$\sigma*$	2.27	1.13	0.05
C7-C9	σ	C5-H13	$\sigma*$	2.19	1.15	0.05
C11-C12	σ	C10-C11	$\sigma*$	2.19	1.32	0.05
O3-C12	σ	O3-C12	$\sigma*$	2.13	1.52	0.05
C10-C11	σ	C4-C6	$\sigma*$	2.08	1.30	0.05
C4-C10	σ	C6-C8	$\sigma*$	2.07	1.25	0.05
C11-C12	σ	O2-H20	$\sigma*$	2.05	1.07	0.04
C7-C9	σ	C8-H16	$\sigma*$	2.03	1.16	0.04
C4-C6	σ	C10-C11	$\sigma*$	1.97	1.34	0.05
C4-C10	σ	C11-C12	$\sigma*$	1.96	1.12	0.04
C4-C10	σ	C5-C7	$\sigma*$	1.88	1.25	0.04
C10-C11	σ	C11-C12	$\sigma*$	1.75	1.19	0.04
C10-C11	σ	C11-H18	$\sigma*$	1.75	1.18	0.04
C11-C12	σ	O3-C12	$\sigma*$	1.74	1.29	0.04
C8-C9	σ	O1-H19	$\sigma*$	1.72	1.10	0.04
C11-H18	σ	C10-C11	$\sigma*$	1.68	1.17	0.04
C10-C11	σ	O2-C12	$\sigma*$	1.55	1.09	0.04
O2-C12	σ	C10-C11	$\sigma*$	1.43	1.56	0.04
C4-C5	σ	C10-H17	$\sigma*$	1.35	1.14	0.04
C5-C7	σ	C7-H15	$\sigma*$	1.31	1.13	0.03
C6-C8	σ	C8-H16	$\sigma*$	1.30	1.15	0.04
O3	LP(1)	O2-C12	$\sigma*$	1.30	1.03	0.03
O1-C9	σ	C6-C8	$\sigma*$	1.28	1.52	0.04
C10-C11	σ	C10-H17	$\sigma*$	1.16	1.18	0.03
O1-C9	σ	C5-C7	$\sigma*$	1.09	1.51	0.04
C10-H17	σ	C10-C11	$\sigma*$	1.08	1.16	0.03
C8-H16	σ	O1-C9	$\sigma*$	1.00	0.86	0.03
