# Supplementary material for: Identification of potent inhibitors of HDAC2 from herbal products for the treatment of colon cancer: Molecular docking, molecular dynamics simulation, MM/GBSA calculations, DFT studies, and pharmacokinetic analysis
Source: PLoS One. 2024 Jul 22;19(7):e0307501. doi: 10.1371/journal.pone.0307501 (PMC11262678; doi:10.1371/journal.pone.0307501)
Supplement: S4 Table — (PDF) [file pone.0307501.s012.pdf]

S4 Table: Results of ADME and toxicity parameters for caffeic acid and p-coumaric acid

(a) Lipinski's rule of 5 parameters for caffeic acid and p-coumaric acid

Particulars	Caffeic acid	p-Coumaric acid
Molecular weight (g/mol or Da)	180.16	164.16
No. of H-bond acceptors	4	3
No. of H-bond donors	3	2
Octanol/water partition coefficient (logP)	1.20	1.49

(b) Some other ADME parameters for caffeic acid and p-coumaric acid

Particulars	Caffeic acid	p-Coumaric acid
Estimated solubility of water (Log S)	-1.89	-2.02
TPSA ($Å^2$)	77.76	57.53
Human Intestinal Absorption (HIA)	0.9739	0.9959
Blood Brain Barrier (BBB)	-0.6500	-0.7750

(c) Toxicity prediction of caffeic acid and p-coumaric acid with probability

Particulars	Toxicity Prediction (Probability) for CA	Toxicity Prediction (Probability) for pCA
Hepatotoxicity	Inactive (0.57)	Inactive (0.51)
Carcinogenicity	Active (0.78)	Active (0.50)
Immunotoxicity	Inactive (0.50)	Inactive (0.91)
Mutagenicity	Inactive (0.98)	Inactive (0.93)
Cytotoxicity	Inactive (0.86)	Inactive (0.81)

(d) LD50 value for toxicity prediction of caffeic acid and p-coumaric acid

Particulars	Caffeic acid	p-Coumaric acid
Predicted LD50	2980 mg/kg	2850 mg/kg
