# Supplementary material for: Identification of potent inhibitors of HDAC2 from herbal products for the treatment of colon cancer: Molecular docking, molecular dynamics simulation, MM/GBSA calculations, DFT studies, and pharmacokinetic analysis
Source: PLoS One. 2024 Jul 22;19(7):e0307501. doi: 10.1371/journal.pone.0307501 (PMC11262678; doi:10.1371/journal.pone.0307501)
Supplement: Attachment — Submitted filename: Response to Reviewer 3.pdf [file pone.0307501.s015.pdf]

Reviewer 3:

We appreciate Reviewer 3's insightful remarks and have addressed your concerns as follows:

Overall, the manuscript has tried to use the most available computational methods to study the two compounds. However, there are many nuances where it could have been more helpful and exciting to add to the current trend in computer-aided drug design, where a few different scaffolds were studied and then compared based on the difference in their molecular features. The readers are just introduced to two highly similar caffeic acid and p-coumaric acid, which perform comparably across all quantifications as expected. I am concerned about the lack of experimental validation, particularly because caffeic acid is more reactive due to the presence of two hydroxyl groups, making it susceptible to oxidation and other chemical reactions. However, this may be helpful as it will help scavenge all ROS species in the stressed cells. But could it cause potential cytotoxicity and further damage? The Bioavailability of caffeic acid has also been questioned recently due to its rapid metabolism. The overall comparison of both compounds in the text, with similar values all along, lacks a strong agreement with caffeic acid being a strong compound to treat cancer.

Response: Thank you for the comments and suggestions. We found that caffeic acid has different effects on the expression of genes related to apoptosis and the induction of apoptosis in treated cancer cells. Specifically, CA being able to affect gene expression and induce apoptosis in cancer cells suggests that it has cytotoxic properties. However, it is crucial that the cytotoxic effects are selective for cancer cells and do not significantly harm normal, healthy cells. [https://doi.org/10.22038%2FAJP.2019.13475]

Further, caffeic acid supplementation improves clinical signs, colonic histopathological score, and colon permeability. Caffeic acid alters gene expression of inflammatory cytokines in the colon. [https://doi.org/10.3389/fmicb.2021.784211]

Recently, to enhance the bioavalibility of caffeic acid, some auxiliary substances, Poloxamer 407 and β-cyclodextrin have been found to enhance the aqueous solubility of caffeic acid and improve its dissolution kinetics. [https://doi.org/10.3390%2Ffoods12101993]
