# Supplementary material for: Identification of potent inhibitors of HDAC2 from herbal products for the treatment of colon cancer: Molecular docking, molecular dynamics simulation, MM/GBSA calculations, DFT studies, and pharmacokinetic analysis
Source: PLoS One. 2024 Jul 22;19(7):e0307501. doi: 10.1371/journal.pone.0307501 (PMC11262678; doi:10.1371/journal.pone.0307501)
Supplement: Attachment — Submitted filename: Comments.docx [file pone.0307501.s013.docx]

The manuscript presents a computational analysis of phytocompounds with anticancer properties by inhibiting HDAC2. The analysis includes docking, MD simulation, MM/GBSA calculations, DFT studies, and pharmacokinetic studies. The manuscript is well-written, and I recommend publishing it with the following revisions:

1. The authors mentioned that among the seventeen phytochemicals, the molecules caffeic acid and p-coumaric acid showed high binding efficacy with HDAC2. However, upon reviewing Table 1S, I noticed that Epipodophyllotoxin and Ferulic acid also showed the same binding efficacy with p-coumaric acid. Could the authors explain why they proceeded with only the first two molecules? Furthermore, based on what criteria did the authors decide not to proceed with Epipodophyllotoxin and Ferulic acid for other computational analyses?

2. The authors should discuss the stability of the observed interactions in the docked pose between the ligands and HDAC2 in the MD simulation. Additionally, it would be beneficial to explain how long these interactions sustained during the simulation period.
