# Supplementary material for: Identification of potent inhibitors of HDAC2 from herbal products for the treatment of colon cancer: Molecular docking, molecular dynamics simulation, MM/GBSA calculations, DFT studies, and pharmacokinetic analysis
Source: PLoS One. 2024 Jul 22;19(7):e0307501. doi: 10.1371/journal.pone.0307501 (PMC11262678; doi:10.1371/journal.pone.0307501)
Supplement: Attachment — Submitted filename: ReviewPLOS-SA-05-06-24.docx [file pone.0307501.s014.docx]

Review PLOS

**Identification of potent inhibitors of HDAC2 from herbal products for the treatment of colon cancer: Molecular docking, molecular dynamics simulation, MM/GBSA calculations, DFT studies, and pharmacokinetic analysis**

*Khanal et al. conducted a study investigating the potential of selected anticancer phytocompounds as HDAC2 inhibitors. Docking simulations revealed that caffeic and p-coumaric acid compounds strongly bound to the HDAC2 enzyme, and MM/GBSA calculations indicated stable protein-ligand interactions within the complexes. Overall, these compounds demonstrated favorable physicochemical and pharmacokinetic properties without toxicity. Notably, the authors claim that they displayed promise as drugs for colon cancer, with caffeic acid being the more optimal choice compared to p-coumaric acid.*

I have several comments to improve the manuscript, as detailed below:

Introduction :

a. In the introduction, highlight a few previous studies illustrating the importance of computational tools in studying anticancer, specifically plant-derived phenolic compounds.
b. Describe the secondary structure composition of HDAC2 as the author talks about the importance of the residues in the binding site. Where does the active site lie?

2. Section 2.1 Molecular docking :
   a. *The active amino acid numbering of the protein was compared between RCSB PDB and SWISS-MODEL, revealing differences in the numbering sequence*
      i. What does the author mean by the difference in the number sequence and the number of missing residues, and in which region of the protein?
      ii. The total number of residues in the protein should be mentioned.
      iii. Highlight the residues missing in the crystal structure
      iv. Which template was used as a template to build the homology model
   b. *The bioactive molecules having anticancer properties were chosen from the literature*
      i. How many compounds were incorporated? Mention the dataset here.
      ii. How were the anticancer compounds filtered from PubChem? What was the rationale behind creating the data? Describe a particular scaffold to add to the information.
   c. Where is the grid box located? The author should highlight and describe a specific region/active site. Was the whole protein used for docking? Clarify.
3. Section 2.1 Molecular Dynamics simulations :
   a. Which force field was employed for the small molecules?
   b. Mention the length of the total simulation.
   c. The strength of NaCl used to neutralize the system.
   d. How was the apo system prepared?
   e. Are any terminal patches applied to the protein to neutralize them?
   f. Was the crystal water retained during the simulations?
   g. How were the results from MD simulations analyzed? Any clustering method used to study the docked complexes?
4. MM/GBSA approach
   a. What are the main contributors to the common binding energy across various interactions, specifically in GbindvdW, GbindLipo, and GbindCoulomb energies?
   b. Does MM/GBSA calculation consider the water molecule in the active site? If the authors had tried these calculations without crystal water and had compared the results, would it have been worse if the water had been included?
   c. How does one validate the precision of data from MM/GBSA calculation while comparing two compounds? If employed, the authors should briefly describe a method.
   d. How do the authors address the statistical convergence of results from MM/GBSA?
   e. Were any rotamer search techniques involved during free energy estimation?
5. Results: Molecular dynamics
   i. The authors should elaborate on the docked ligand's RMSD compared to the crystal structures and discuss any deviations in interactions with the active site residues. The simulation clustered data also gives a more detailed insight into which bonds stay stable during the simulation.
   ii. How many simulation replicates were run for the analysis of each compound? If the authors ran only one, how would they emphasize and ensure the simulations were converged?
   iii. *The average Rg values of Cα atoms for the apo form (1.99 ± 0.01 nm), CA-HDAC2 complex (1.98 ± 0.01 nm), and the pCA-HDAC2 complex (1.98 ± 0.01 nm) are nearly identical. The HDAC2 protein is significantly stable in the presence of CA and pCA molecules during the simulation period with low average values of Rg.*
        1. The authors claim that HDAC2 protein is more stable in the presence of CA than pCA. However, the Rg values are exactly identical. Even when compared to apo, the protein does not show much instability in the absence of either of these compounds. This rationale seems weak, and the authors should suggest a stronger point to comment on the stability of the complex.
6. Results: MM/GBSA
   a. *Within the active residues of HDAC2, MET24 has the lowest binding energy of -2.44 ± 1.00 kcal/mol in CA-HDAC2 complex and* ***TYR18*** *has the lowest binding energy of -1.73 ± 0.75 kcal/mol in pCA-HDAC2 complex*
      i. Inconsistency is residue annotation “Tyr18.”
   b. *The average value of RMSF for apo form is 0.72 ± 0.43 A and ˚ for* ***CA-HDAC2 and CA-HDAC2*** *complexes are 0.83 ± 0.36 A and 0.83 ˚ ± 0.51 A, respectively.*
      i. Did the authors mean CA-HDAC2 and pCA-HDAC2?
7. Grammer inconsistencies in the text.
8. Figure 1s Atomic notation
9. Figure 2: The figures' quality is hazy, so they should be saved in TIFF format to achieve the maximum quality for publication images.
10. Figure 3, panel A, RMSD of complex / ligand/ protein backbone? Kindly update the label on the y-axis appropriately.
11. Figure 3, panel E, SASA of what needs to be mentioned and updated.
12. Figure 4, Describe the difference in RMSF between all three system set-ups at residue number ~198 (spike region ~5 Angstrom). The authors should elaborate on where this residue lies and why we see a significantly low RMSF in CA during the simulations.
13. A figure illustrating the alignment of the docked compound to the sampled conformations from MD simulation and crystal structure could be meaningful.

Overall, the manuscript has tried to use the most available computational methods to study the two compounds. But there are many nuances where it could have been more helpful and exciting to add to the current trend in Computer-aided drug design where a few different scaffolds were studied and then compared based on the difference in their molecular features. The readers are just introduced to two highly similar caffeic acid and p-coumaric acid, which perform comparably across all quantifications as expected. I have an overall concern about the lack of experimental validation, particularly because caffeic acid is more reactive due to the presence of two hydroxyl groups, which makes it susceptible to oxidation and other chemical reactions. However, this may be helpful as it will help scavenge all ROS species in the stressed cells. But could it cause potential cytotoxicity and further damage? The Bioavailability of caffeic acid has also been in question recently due to its rapid metabolism. The overall comparison of both compounds in the text, with similar values all along, lacks a strong agreement with caffeic acid being a strong compound to treat cancer.
